# Positive Effects of Three-Dimensional Collagen-Based Matrices on the Behavior of Osteoprogenitors

**DOI:** 10.3389/fbioe.2021.708830

**Published:** 2021-07-21

**Authors:** Zhikai Lin, Cristina Nica, Anton Sculean, Maria B. Asparuhova

**Affiliations:** ^1^ Laboratory of Oral Cell Biology, Dental Research Center, School of Dental Medicine, University of Bern, Bern, Switzerland; ^2^ Department of Periodontology, School of Dental Medicine, University of Bern, Bern, Switzerland; ^3^ Department of Periodontology, Shanghai Ninth People’s Hospital, Shanghai Jiao Tong University School of Medicine, College of Stomatology, Shanghai Jiao Tong University, National Center for Stomatology, National Clinical Research Center for Oral Diseases, Shanghai Key Laboratory of Stomatology, Shanghai, China

**Keywords:** three-dimensional biomaterials, xenografts, bone regeneration, periodontal regeneration, osteogenesis, growth factors, gene expression, transcription

## Abstract

Recent research has demonstrated that reinforced three-dimensional (3D) collagen matrices can provide a stable scaffold for restoring the lost volume of a deficient alveolar bone. In the present study, we aimed to comparatively investigate the migratory, adhesive, proliferative, and differentiation potential of mesenchymal stromal ST2 and pre-osteoblastic MC3T3-E1 cells in response to four 3D collagen-based matrices. Dried acellular dermal matrix (DADM), hydrated acellular dermal matrix (HADM), non-crosslinked collagen matrix (NCM), and crosslinked collagen matrix (CCM) did all enhance the motility of the osteoprogenitor cells. Compared to DADM and NCM, HADM and CCM triggered stronger migratory response. While cells grown on DADM and NCM demonstrated proliferative rates comparable to control cells grown in the absence of a biomaterial, cells grown on HADM and CCM proliferated significantly faster. The pro-proliferative effects of the two matrices were supported by upregulated expression of genes regulating cell division. Increased expression of genes encoding the adhesive molecules fibronectin, vinculin, CD44 antigen, and the intracellular adhesive molecule-1 was detected in cells grown on each of the scaffolds, suggesting excellent adhesive properties of the investigated biomaterials. In contrast to genes encoding the bone matrix proteins collagen type I (Col1a1) and osteopontin (Spp1) induced by all matrices, the expression of the osteogenic differentiation markers Runx2, Alpl, Dlx5, Ibsp, Bglap2, and Phex was significantly increased in cells grown on HADM and CCM only. Short/clinically relevant pre-coating of the 3D biomaterials with enamel matrix derivative (EMD) or recombinant bone morphogenetic protein-2 (rBMP-2) significantly boosted the osteogenic differentiation of both osteoprogenitor lines on all matrices, including DADM and NCM, indicating that EMD and BMP-2 retained their biological activity after being released from the matrices. Whereas EMD triggered the expression of all osteogenesis-related genes, rBMP-2 upregulated early, intermediate, and late osteogenic differentiation markers except for Col1a1 and Spp1. Altogether, our results support favorable influence of HADM and CCM on the recruitment, growth, and osteogenic differentiation of the osteoprogenitor cell types. Furthermore, our data strongly support the biofunctionalization of the collagen-based matrices with EMD or rBMP-2 as a potential treatment modality for bone defects in the clinical practice.

## Introduction

Augmentation of bone defects remains a major challenge in reconstructive orthopedic, periodontal, and maxillofacial surgeries. Bone atrophy often results from trauma, infection, neoplasm, congenital disorder, or tooth extraction. Periodontal disease, endodontic lesions, severe tooth decay, or fracture can necessitate a tooth extraction. The majority of treatment modalities include the use of autogenous bone as a gold standard as well as xenogenic, allogenic, or alloplastic bone substitutes (Doonquah et al., 2021). The ideal biomaterial for the purpose of bone regeneration should be biocompatible, volume stable (space-making), osteoconductive, and osteoinductive (Yamada and Egusa, 2018). Furthermore, it should possess a predictable pattern of biodegradability, be easy for manufacturing and handling, and highly cost effective. As a primary component of the bone matrix that plays a role in numerous cellular processes, collagen appears a potential candidate for the design of three-dimensional (3D) scaffolds for bone regeneration (Pawelec et al., 2016; Pabst and Kämmerer, 2020). However, it is well known that collagen biomaterials have high biodegradability and low mechanical strength. Therefore, attempts have been made to improve the collagen scaffolds for bone tissue engineering, e.g., collagen-based composite scaffolds with bioceramic, carbon, and polymer components have been proposed (Zhang et al., 2018). Furthermore, non-enzymatic or enzymatic crosslinking procedures have been utilized in order to reduce the naturally high biodegradability and to increase the mechanical stability of the collagen matrices by establishing intermolecular bonds (Adamiak and Sionkowska, 2020). The combination of 3D collagen matrices with bone substitute materials (Basudan et al., 2016; Papi et al., 2021) or bioactive substances (Herford and Cicciù, 2012; Herford et al., 2012; Ramseier et al., 2012; Jin and Giannobile, 2014; Edelmayer et al., 2020; França-Grohmann et al., 2020) for the repair of periodontal hard tissue loss and severe alveolar ridge deficiencies have appeared as promising strategies.

In a recent study, we have demonstrated that four commercially available 3D collagen-based matrices of porcine origin can be efficiently loaded with enamel matrix derivative (EMD) or recombinant growth factors such as the transforming growth factor-β1 (TGF-β1), fibroblast growth factor-2 (FGF-2), platelet-derived growth factor-BB (PDGF-BB), growth and differentiation factor-5 (GDF-5), or the bone morphogenetic protein-2 (BMP-2) (Nica et al., 2020). Except for recombinant GDF-5, the loading efficiency of the investigated growth factors was close to 100%. Furthermore, the matrices have exhibited sustained growth factor release over 13 days with kinetics that will likely favor the long-term tissue regeneration following surgical reconstructive periodontal therapies. We have further demonstrated that the investigated collagen-based matrices successfully promote migration, adhesion, and proliferation of cell types involved in oral soft tissue regeneration, namely primary human oral fibroblasts and periodontal ligament cells (Lin et al., 2020). The current study extends the *in vitro* investigations on the four 3D matrices in relation to their ability to trigger osteogenic differentiation.

Osteogenesis is a complex multistep process that requires a biomaterial with excellent physicochemical and biological properties in order to support the migration, attachment, proliferation, and differentiation of osteoprogenitor cells at the defect site. The biomaterials under investigation in the current study were selected based on their commercial availability, easy supply, excellent characteristics declared by the manufacturers, and limited (if any) data for their potential utilization in supporting bone regeneration. Therefore, one of the examined biomaterials is a dry-supplied acellular dermal matrix (mucoderm®; botiss biomaterials GmbH, Zossen, Germany), labelled DADM. *In vitro* and *in vivo* cell-matrix interaction studies have shown that DADM supports the metabolic activity and proliferation of various cell types including osteoblasts (Pabst et al., 2014). Furthermore, a successful biofunctionalization of DADM with EMD or platelet-rich fibrin have positively influenced the behavior of primary human endothelial cells *in vitro* (Park et al., 2018; Blatt et al., 2020) as well as the angiogenesis *in vivo* (Blatt et al., 2020). A novel tissue-engineered acellular dermal matrix provided in a hydrated form, labeled HADM (NovoMatrix™ Reconstructive Tissue Matrix; BioHorizons, Birmingham, AL, United States), has shown consistent favorable effects on the behavior of primary human oral fibroblasts and periodontal ligament cells *in vitro* (Lin et al., 2020) as well as in treating gingival recession defects in an *in vivo* animal model (Suárez-López Del Amo et al., 2019). The third examined xenograft is a non-crosslinked collagen matrix, labeled NCM (Geistlich Mucograft®; Geistlich, Wolhusen, Switzerland) and composed of native collagen types I and III (Nevins et al., 2003; Ghanaati et al., 2011). Interestingly, the addition of PDGF-BB to the NCM was shown to accelerate soft tissue healing and promote bone formation in bilateral mandibular alveolar defects of a minipig model (Herford and Cicciù, 2012; Herford et al., 2012). Furthermore, similar to the effects of adsorbed BMP-7, vascular endothelial growth factor and PDGF, the stromal-derived factor-1 as a potent chemoattractant of circulating stem cells was successfully adsorbed on NCM, resulting in improved bone healing at calvarial critical-sized defects in a pre-clinical murine model (Jin and Giannobile, 2014). The final forth xenograft included in the study is a novel ribose-crosslinked collagen matrix, labeled CCM (Ossix® Volumax; Datum Dental Ltd., Lod, Israel). This thick resorbable collagen scaffold, reinforced by a novel proprietary crosslinking technology (Glymatrix®), was able to restore the lost volume of a deficient ridge between existing teeth (Smidt et al., 2019). The authors of the study commented that the augmentation procedure using CCM was simpler to perform compared to procedures with bone substitute materials and/or an interpositional connective tissue graft harvested from a remote donor site. Reinforced collagen membranes of the same product family have been shown to induce bone regeneration in critical-sized alveolar ridge defects in a dog model (Zubery et al., 2007) as well as in humans with direct mineral apposition on the glycated collagen (Zubery et al., 2008). On a cellular level, the glycated collagen membrane promoted the attachment and proliferation of human periodontal ligament fibroblasts and human SaOs-2 osteoblasts (Rothamel et al., 2004). However, thorough analyses of the responses of osteoprogenitor cells to each of the listed 3D collagen-based matrices are entirely lacking.

The aim of the present study was to investigate the migratory, proliferative, and adhesive properties as well as the osteogenic differentiation potential of mesenchymal stromal and pre-osteoblastic cells cultured on each of the four collagen-based matrices. The study further aimed to investigate whether the biological activity of EMD or BMP-2 can be transferred onto the biomaterials *in vitro*, leading to enhanced osteogenic properties of the two cell types.

## Materials and Methods

### Cell Culture and 3D Xenogenic Collagen-Based Matrices

Two types of osteoprogenitors of mouse origin were used throughout the study: bone marrow-derived mesenchymal stromal ST2 cells were obtained from the RIKEN Cell Bank (Tsukuba, Japan) and calvaria-derived pre-osteoblastic MC3T3-E1 cells were obtained from the ECACC collection (Sigma, Buchs, Switzerland). Both cell lines were characterized as osteoprogenitors by well-documented past (Franceschi and Iyer, 1992; Quarles et al., 1992; Franceschi et al., 1994; Torii et al., 1996; Otsuka et al., 1999) and recent studies (Cui et al., 2016; Parisi et al., 2021), and considered good models for studying osteogenesis *in vitro*. Both lines were grown in Dulbecco’s Modified Eagle Medium (DMEM) supplemented with 10% fetal calf serum (FCS; Invitrogen, Zug, Switzerland) and 1% antibiotics/antimycotics (ThermoFisher Scientific, Basel, Switzerland). Cells were starved in 0.3% FCS/DMEM for 24 h before their culturing under experimental conditions.

DADM [kindly provided by botiss biomaterials GmbH (Berlin, Germany)], HADM [kindly provided by Camlog Biotechnologies GmbH (Basel, Switzerland)], NCM, and CCM [kindly provided by Datum Dental Ltd., (Lod, Israel)] were cut sterile into 10 × 10 mm pieces, washed in serum-free DMEM for 10 min and placed on the bottom of 24-well ultra-low attachment plates (Corning, NY, United States). Cells grown in tissue culture-treated 24-well plates (Greiner Bio-One, St. Gallen, Switzerland) in the absence of a biomaterial were used as control (Ctrl).

In some cases, the collagen matrices were coated for 10 min at room temperature with 1 mg/ml of EMD (Straumann® Emdogain®; botiss biomaterials GmbH, Zossen, Germany) or 100 ng/ml of recombinant (r) BMP-2 (Peprotech, London, United Kingdom). The EMD and rBMP-2 were diluted in serum-free DMEM from a 10 mg/ml and 10 μg/ml stock solutions, respectively. Collagen-based matrices incubated in serum-free DMEM, in the absence of EMD or rBMP-2, were used as controls. To remove unbound proteins after the 10-min incubation, the collagen matrices were extensively washed in serum-free DMEM for three cycles of 5 min each. The quantities of TGF-β1 [as a measure for the release of EMD (Stähli et al., 2014; Stähli et al., 2016)] and BMP-2 in culture supernatants of cells grown on EMD- or BMP-2-coated collagen matrices, respectively, were determined by using colorimetric enzyme-linked immunosorbent assay (ELISA; R&D Systems, Minneapolis, MN, United States) as described (Nica et al., 2020) and following the manufacturer’s protocol.

For differentiation experiments followed by gene expression analyses, 10% FCS/DMEM medium was supplemented with 50 μg/ml ascorbic acid (Invitrogen) and 2 mM β-glycerophosphate (Invitrogen) as described (Parisi et al., 2021).

### Cell Migration Assay

Cell migration was analyzed by a Boyden chamber assay utilizing ThinCert® transwell polyethylene terephthalate (PET) membrane supports (8 µm pore size; Greiner Bio-One, St. Gallen, Switzerland) as described (Asparuhova et al., 2021). After 24 h of starvation, 3 × 10^4^ cells were cultured in the top insert chamber with 200 µl 0% FCS/DMEM. Each of the collagen matrices was placed in the low chamber with 800 µl 10% FCS/DMEM. Cells were allowed to migrate across the capillary pore PET membrane for 18 h at 37°C before fixation in Shandon™ Formal-Fixx™ (ThermoFisher Scientific), and staining in 0.1% crystal violet solution (Sigma). Images of duplicate inserts were acquired on an Olympus CKX41 microscope using a ProgResCT3 camera. Migration was quantified by using the ImageJ software (Schneider et al., 2012) as described (Gurbuz et al., 2014). Data represent means ± SD from three independent experiments performed with each of the two cell lines.

### Cell Proliferation Assay

Growth rates of ST2 and MC3T3-E1 cells cultured on the collagen-based matrices were determined by trypan blue dye exclusion cell counting performed in a Countess™ II instrument (Invitrogen) according to the manufacturer’s instructions. After 24 h of starvation, 2 × 10^3^ cells/well were plated in 3% FCS/DMEM and allowed to proliferate for 1, 3, and 6 days before staining with 0.4% trypan blue (Invitrogen) solution. The culture media was replaced every 2 days. Data represent means ± SD from four independent experiments performed with each of the two cell lines.

### Gene Expression Analysis

Quantitative reverse transcription-polymerase chain reaction (qRT-PCR) was used to investigate the expression of three groups of genes: 1) proliferative markers (Mybl2, Bub1, Plk1, Mki67, Pcna, Ccne1, Ccnd1, and Ccnb1), 2) adhesive markers (Fn1, Vcl, Cd44, and Icam1), and 3) osteogenesis markers (Col1a1, Spp1, Runx2, Alpl, Dlx5, Ibsp, Bglap2, and Phex) as described (Lin et al., 2020; Parisi et al., 2021).

After 24 h of starvation, 2.5 × 10^5^ cells/well were cultured in 3% FCS/DMEM or osteogenic supplements-containing 10% FCS/DMEM in the absence (control) or in the presence of each of the four collagen matrices. Proliferative or osteogenesis markers, respectively, were analyzed on day 3 post-seeding. In addition, osteogenesis marker gene expression was analyzed in cells grown for 3 days on native/uncoated matrices (control groups) or matrices coated with either EMD or rBMP-2 (test groups), according to the coating procedure described in *Cell Culture and 3D Xenogenic Collagen-Based Matrices*.

For analysis of adhesive marker gene expression, 6 × 10^5^ cells/well were seeded in 10% FCS/DMEM in the absence (control) or in the presence of each of the four matrices and allowed to adhere for 10 h. After removal of the culture medium and before cell lysis, control cells and collagen matrices seeded with cells were extensively rinsed three times in phosphate-buffered solution (PBS) for complete removal of nonadherent cells.

Total RNA from cells of each experimental group was isolated using TRIzol (ThermoFisher Scientific) according to the manufacturer’s instructions. The extracted RNA was additionally purified by using the RNeasy MinElute Cleanup Kit (Qiagen, Basel, Switzerland). RNA, spectrophotometrically quantified on a NanoDrop 2000c instrument (ThermoFisher Scientific), was reverse transcribed using the Applied Biosystems™ High-Capacity cDNA Reverse Transcription Kit (ThermoFisher Scientific). Subsequently, relative transcripts for the above listed genes, normalized to the internal control Gapdh, were quantified using FastStart Universal SYBR Green Master ROX (Roche, Basel, Switzerland) and the primer sequences listed in Supplementary Tables 1–3. Quantitative PCR was carried out in a QuantStudio 3 instrument (Applied Biosystems, Rotkreuz, Switzerland) using a standard thermal cycling profile. The efficiency ∆∆Ct method was used to calculate gene expression levels normalized to Gapdh values and calibrated to values of controls. Samples were run in duplicates. Data represent means ± SD from four independent experiments performed with each of the two cell lines.

### Statistical Analysis

Grouped data is represented by means ± SD. Differences between groups were assessed by one-way analysis of variance (ANOVA) with Tukey’s post-hoc test using GraphPad InStat Software (GraphPad, La Jolla, CA, United States), version 3.05. Significance was indicated using the scale, ***p < 0.001, **p < 0.01, and *p < 0.05.

## Results

### Increased Migratory Potential of Osteoprogenitor Cell Lines Toward Four Different Collagen-Based Matrices

**FIGURE 1:**
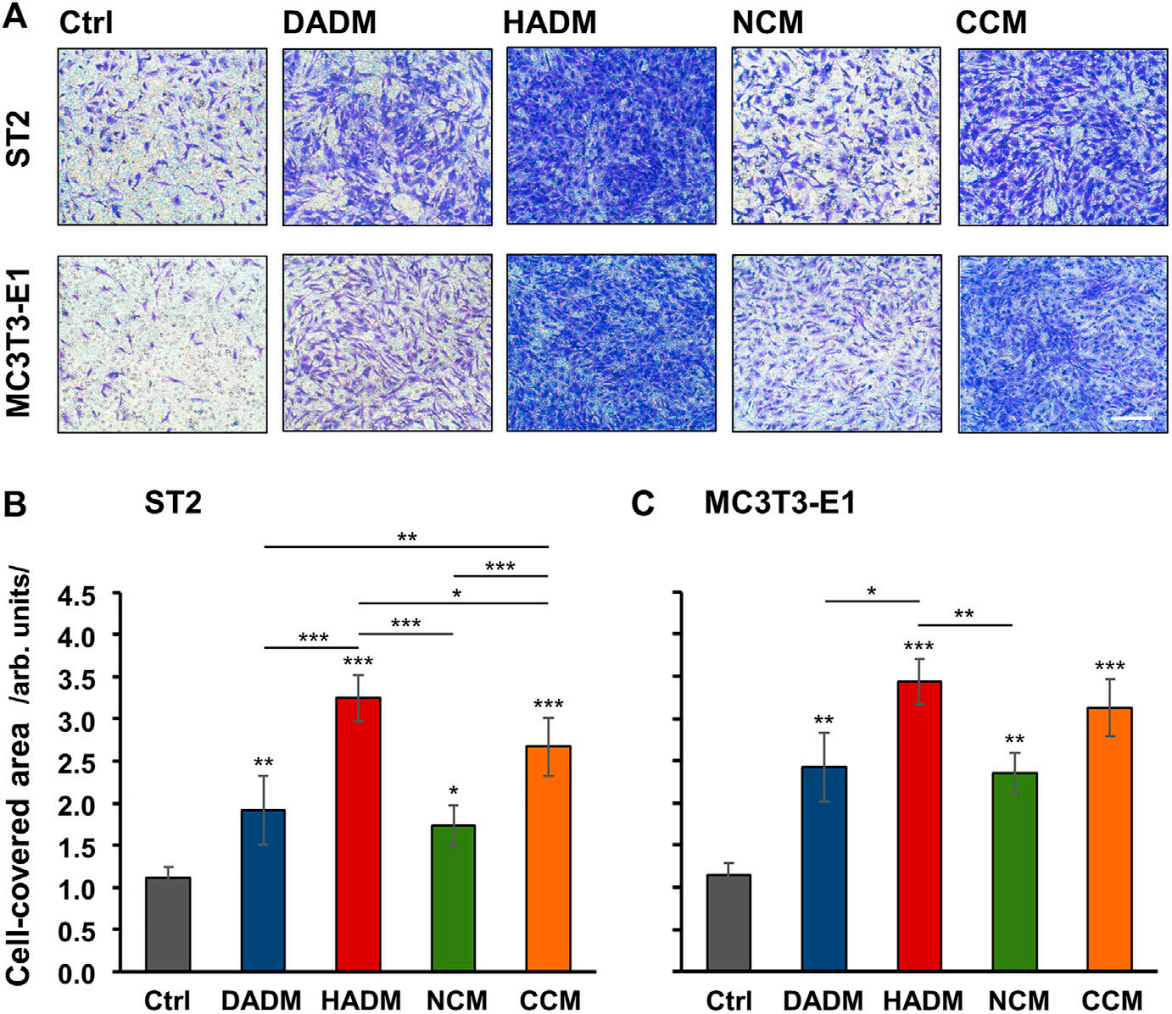
Increased migratory potential of osteoprogenitor cell lines toward four different collagen-based matrices. Migration of mesenchymal stromal ST2 **(A, B)** and pre-osteoblastic MC3T3-E1 **(A, C)** cells toward DADM, HADM, NCM, and CCM matrices was evaluated by a modified Boyden chamber migration assay utilizing ThinCert® transwell PET membrane supports with 8 μm pore size. **(A)** Representative images of fixed and stained cells that have migrated to the lower side of the membrane in each of the experimental groups. Scale bar, 500 μm. **(B, C)** Quantification of the cell migration in the absence (Ctrl) or presence of collagen-based matrices by using the Image J software measuring the area on the lower side of the membrane support covered with migrated cells. Data represent means ± SD from three independent experiments performed with each of the two cell lines. Significant differences to the respective controls unless otherwise indicated, ***p < 0.001, **p < 0.01, *p < 0.05.

Migratory properties of mesenchymal stromal ST2 and pre-osteoblastic MC3T3-E1 cells toward the investigated 3D matrices were examined *in vitro* by using a modified Boyden chamber migration assay. Each of the four matrices caused significant (*p* < 0.05) induction in the migration rate of the two cell lines compared to control cells, where the migration occurred in the absence of a matrix (Figure 1). Compared to control cells, HADM caused the highest (3-fold) and most significant (*p* < 0.001) increase in the migration rate of the two cell lines. By effectiveness, HADM was immediately followed by CCM, which induced strongly significant (*p* < 0.001) cell migration by 2.4-fold in ST2 and 2.7-fold in MC3T3-E1 cells compared to the respective controls.

Compared to DADM and NCM, the pro-migratory properties of HADM and CCM were significantly better pronounced in ST2 cells only (Figure 1B). In MC3T3-E1 cells, HADM triggered significantly higher migration compared to DADM (*p* < 0.05) and NCM (*p* < 0.01) whereas the pro-migratory effect of CCM was stronger but not significantly different than the effect caused by DADM and NCM (Figure 1C). Furthermore, a significant difference in the effects caused by CCM and HADM, in favor of the latter, was observed in ST2 (*p* < 0.05; Figure 1B) but not in MC3T3-E1 (Figure 1C) cells.

### Strongly Induced Proliferation of Osteoprogenitor Cell Lines Grown on the Hydrated Acellular Dermal Matrix and the Ribose-Crosslinked Collagen Matrix

**FIGURE 2:**
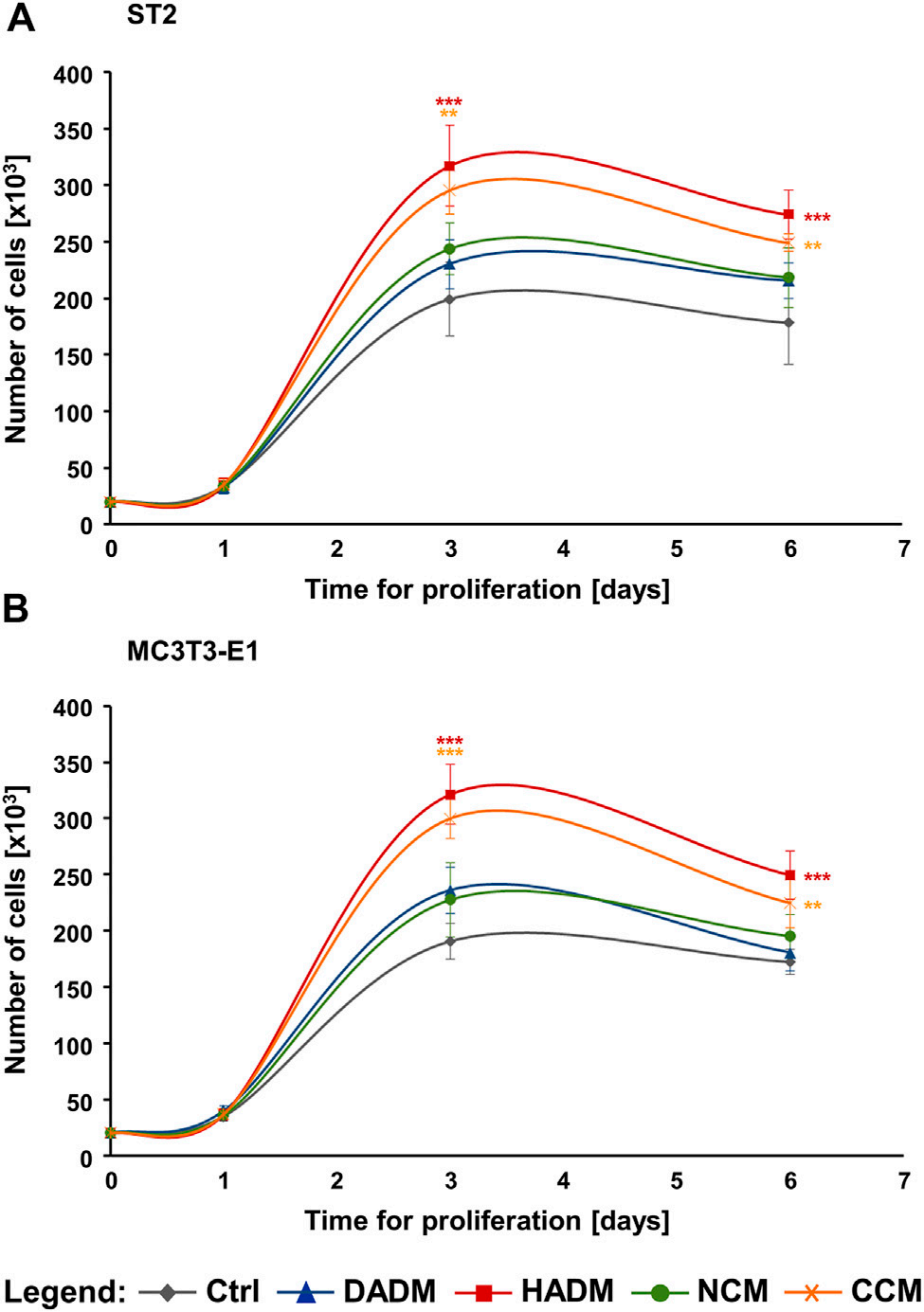
Strongly induced proliferation of osteoprogenitor cell lines grown on HADM and CCM collagen-based matrices. Proliferation rates of ST2 **(A)** and MC3T3-E1 **(B)** cells cultured in the absence of a matrix (Ctrl) or on each of the four (DADM, HADM, NCM, or CCM) collagen-based matrices were assessed by automated trypan blue dye exclusion cell counting. The number of viable cells in each experimental group was determined on day 1, 3, and 6. Data represent means ± SD from three independent experiments performed with each of the two cell lines. Significant differences to control cells at each individual time point, ***p < 0.001, **p < 0.01.

The proliferative rates of ST2 and MC3T3-E1 cells grown on each of the four collagen-based matrices were assessed by trypan blue dye exclusion cell counting performed in a Countess™ II instrument on days 1, 3, and 6 post-seeding. The four matrices remained compact and showed no signs of degradation during the 6-day culture period. After day 1, differences in the growth of the two cell lines on each of the matrices were detected. Both ST2 and MC3T3-E1 cells exhibited significantly higher (>1.5-fold) proliferative rates on HADM and CCM compared to control cells, with *p* < 0.01 between day 3 and 6 (Figure 2). In contrast, ST2 and MC3T3-E1 cells grew slightly but not significantly faster on DADM and NCM compared to the growth of control cells.

In addition, compared to DADM and NCM, the pro-proliferative effect of HADM appeared to be significantly better pronounced in both osteoprogenitor lines between day 3 and 6 (Figures 2A,B). In contrast, CCM did not exhibit a consistently higher effect on the proliferative rate of the two cell lines compared to DADM and NCM. ST2 and MC3T3-E1 cells grew with a significantly higher rate on CCM compared to DADM on day 3 (Figures 2A,B) but only the pre-osteoblastic MC3T3-E1 cells were faster growing on CCM compared to NCM on the same time point (Figure 2B). In the sake of a clearer visualization, symbols for significance are depicted for each collagen matrix tested compared to the control group only.

### Increased Expression of Proliferative Marker Genes in Osteoprogenitor Cells Grown on the Hydrated Acellular Dermal Matrix and the Ribose-Crosslinked Collagen Matrix

**FIGURE 3:**
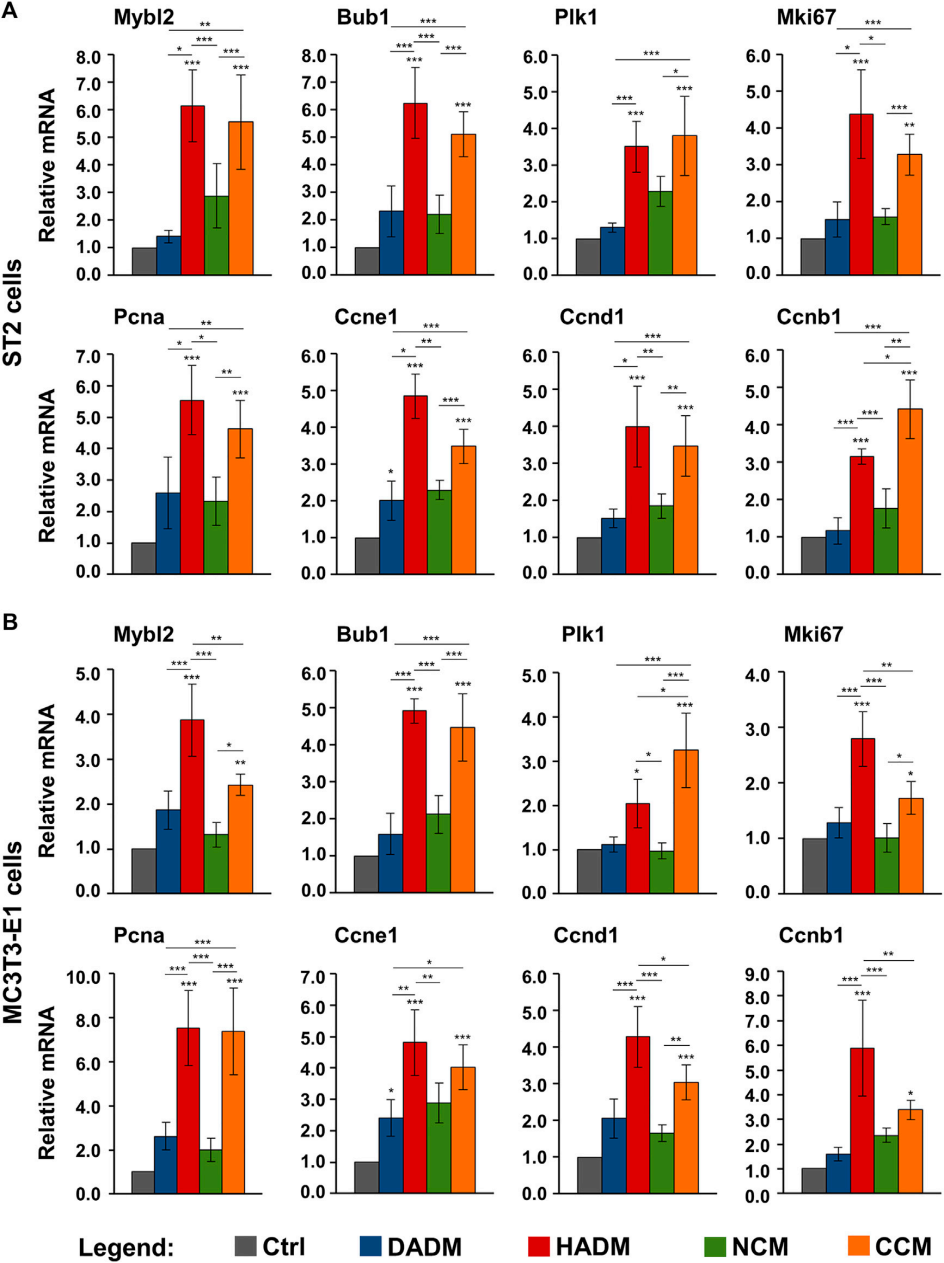
Increased expression of proliferate marker genes in osteoprogenitor cells grown on HADM and CCM collagen-based matrices. ST2 **(A)** and MC3T3-E1 **(B)** cells were grown on DADM, HADM, NCM, or CCM collagen-based matrices for 3 days before total cellular RNA was extracted and analyzed for the expression of Mybl2, Bub1, Plk1, Mki67, Pcna, Ccne1, Ccnd1, and Ccnb1 proliferative marker genes by qRT-PCR. Controls (Ctrl) represent cells of each cell type grown in the absence of a collagen matrix. Values normalized to Gapdh are expressed relative to the values of control cells. Data represent means ± SD from four independent experiments performed with each of the two cell lines. Significant differences to the respective controls unless otherwise indicated, ***p < 0.001, **p < 0.01, *p < 0.05.

To confirm the increased proliferative rates of osteoprogenitors grown on HADM and CCM matrices and to investigate further, how these two collagen-based scaffolds exhibit their effect on the growth of the ST2 and MC3T3-E1 cells, we performed a screen for the expression of genes involved in the regulation of the cell cycle progression (Whitfield et al., 2006). These are Mybl2 encoding the Myb-related protein B, Bub1 encoding a mitotic checkpoint serine/threonine-protein kinase, Plk1 encoding the polo-like kinase 1, Mki67 encoding the Ki-67 proliferative marker, Pcna encoding the proliferating cell nuclear antigen, and Ccne1, Ccnd1, and Ccnb1 encoding cyclin-E1, -D1, and -B1, respectively. The expression of the listed proliferative markers was analyzed by qRT-PCR. In agreement with the proliferation data as seen in Figure 2, on day 3, we observed a general trend of induced expression of the majority of the investigated proliferative marker genes in ST2 and MC3T3-E1 cells cultured on each of the collagen-based matrices compared to the expression levels detected in control cells (Figure 3). However, among the four investigated matrices, only HADM and CCM caused statistically significant (*p* < 0.05) upregulation of all proliferative markers in both osteoprogenitor cell lines, in the range of 2.0–7.5-fold, compared to control cells cultured in the absence of a matrix. In both cell lines, the Ccne1 was the only gene that appeared significantly (*p* < 0.05) induced in cells grown on DADM compared to control cells (Figures 3A,B).

Interestingly, two cell type-specific differences in the effects of the investigated matrices on the proliferative marker gene expression were detected. In the ST2 cells, the HADM and CCM matrices performed significantly (*p* < 0.01) better than DADM and NCM matrices (Figure 3A). The only exception was seen for the Plk1 gene expression that did not significantly differ between cells cultured on HADM and NCM. Moreover, except for Ccnb1 gene, which was significantly (*p* < 0.05) stronger induced in ST2 cells cultured on CCM than on HADM matrix, no further differences in the effects caused by HADM and CCM were observed and the two matrices performed equally well. In contrast to ST2 cells, four out of the eight investigated proliferative marker genes were significantly (*p* < 0.05) stronger upregulated in MC3T3-E1 cells cultured on HADM compared to CCM (Figure 3B). These were Mybl2, Mki67, Ccnd1, and Ccnb1. In these four cases, the difference in the effect caused by CCM and HADM, in favour of the latter, was also accompanied by no significant difference in the performance between CCM and DADM or CCM and NCM (in the case of Ccnb1). Plk1 was the only gene that was significantly (*p* < 0.05) better expressed in MC3T3-E1 cells cultured on CCM compared to its expression in cells cultured on HADM and respectively, no difference in the effects caused by HADM and DADM were observed.

In summary, the increased expression of genes regulating the cell cycle progression in the two osteoprogenitor cell lines grown on HADM and CCM matrices supports at least in part the strong pro-proliferative effect of HADM and CCM.

### Increased Expression of Adhesive Marker Genes in Osteoprogenitor Cells Grown on Four Different Collagen-Based Matrices

Cellular adhesion precedes functional differentiation of osteoprogenitors (Biggs and Dalby, 2010). Therefore, we performed a screen for the expression of several adhesive marker genes, such as Fn1, Vcl, Cd44, and Icam1, in ST2 and MC3T3-E1 cells grown on the collagen-based scaffolds. Fn1 gene encodes fibronectin, which is a ubiquitously expressed, non-collagenous extracellular matrix (ECM) protein with a major role in regulating cell adhesion and differentiation (Newby et al., 2020). Vcl encodes vinculin, which is an essential component of the focal adhesions and associated with both cell-cell and cell-matrix interactions (Bays and DeMali, 2017). Cd44 and Icam1 encode the CD44 antigen and the intercellular adhesion molecule-1, respectively. Both are cell surface glycoprotein receptors that promote not only cell-cell and cell-matrix adhesion but also the migration and retention of inflammatory cells to various tissues (Hosokawa et al., 2006; Leonardi et al., 2006; Lucarini et al., 2009).

**FIGURE 4:**
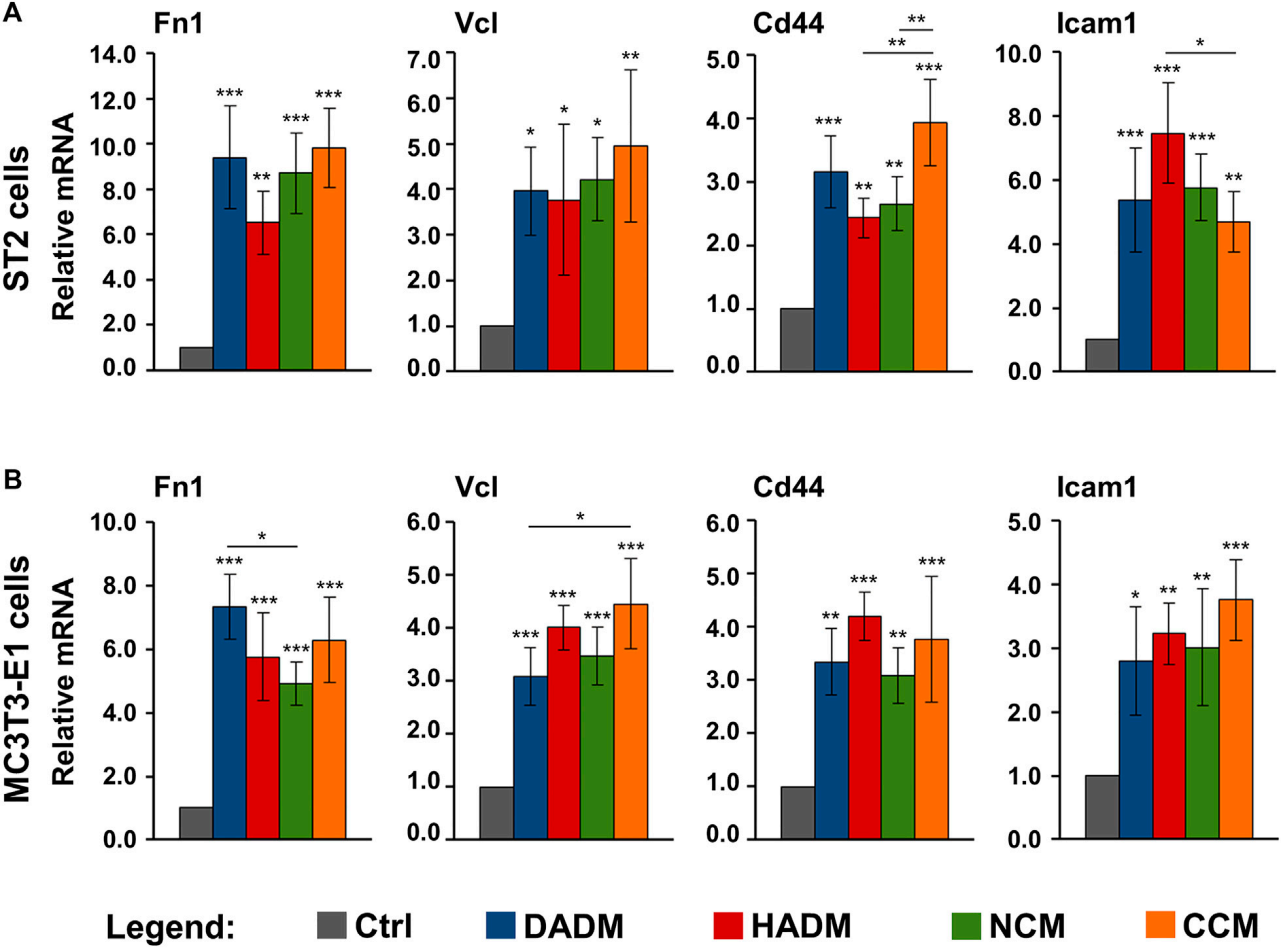
Increased expression of adhesive marker genes in osteoprogenitor cells grown on four different collagen-based matrices. ST2 **(A)** and MC3T3-E1 **(B)** cells were cultured in the absence of a matrix (Ctrl) or on DADM, HADM, NCM, or CCM collagen-based matrices for 10 h followed by an extensive wash for complete removal of nonadherent cells. Subsequently, total cellular RNA was isolated, purified, and analyzed for the expression of Fn1, Vcl, Cd44, and Icam1 adhesive markers by qRT-PCR. Values normalized to Gapdh are expressed relative to the values of control cells. Data represent means ± SD from four independent experiments performed with each of the two cell lines. Significant differences to the respective controls unless otherwise indicated, ***p < 0.001, **p < 0.01, *p < 0.05.

Quantification of the expression of the above listed adhesive marker genes by means of qRT-PCR revealed a significant (*p* < 0.05) induction of all four mRNA levels in both ST2 (Figure 4A) and MC3T3-E1 (Figure 4B) cells grown on each of the four collagen matrices above the expression levels detected in control cells. The observed upregulation in the expression of Fn1, Vcl, Cd44, and Icam1 was comparable in both osteprogenitor lines and in the range of 2.4–9.8-fold (Figures 4A,B). Cd44 was characterized with a significantly (*p* < 0.01) higher expression in ST2 cells grown on CCM compared to its expression in ST2 cells grown on HADM and NCM (Figure 4A). On contrary, HADM caused a significantly (*p* < 0.05) higher upregulation of Icam1 expression than CCM in the ST2 cells. Isolated cases of a more potent effect of DADM compared to NCM as well as of CCM compared to DADM on the induction of Fn1 and Vcl in MC3T3-E1 cells, respectively, were also observed (Figure 4B).

Taken together, our data demonstrate a potent pro-adhesive capacity of the four collagen matrices with no clear trend for a difference in their potency.

### Increased Expression of Osteogenic Differentiation Markers in Osteoprogenitor Cells Grown on the Hydrated Acellular Dermal Matrix and the Ribose-Crosslinked Collagen Matrix

As a next step, a screen for the expression of osteogenesis-related genes was performed. Therefore, ST2 and MC3T3-E1 cells grown on each of the investigated scaffolds were analyzed for the expression of 1) genes encoding bone matrix proteins such as collagen type I (Col1a1) and osteopontin (also known as secreted phosphoprotein 1, Spp1); 2) genes encoding early osteogenic markers such as the runt-related transcription factor 2 (Runx2) and alkaline phosphatase (Alpl); and 3) genes encoding intermediate and late osteogenic markers such as distal-less homeobox 5 (Dlx5), integrin-binding sialoprotein (Ibsp), osteocalcin (or bone gamma-carboxyglutamate protein 2, Bglap2), and phosphate regulating endopeptidase homolog, X-linked (Phex).

**FIGURE 5:**
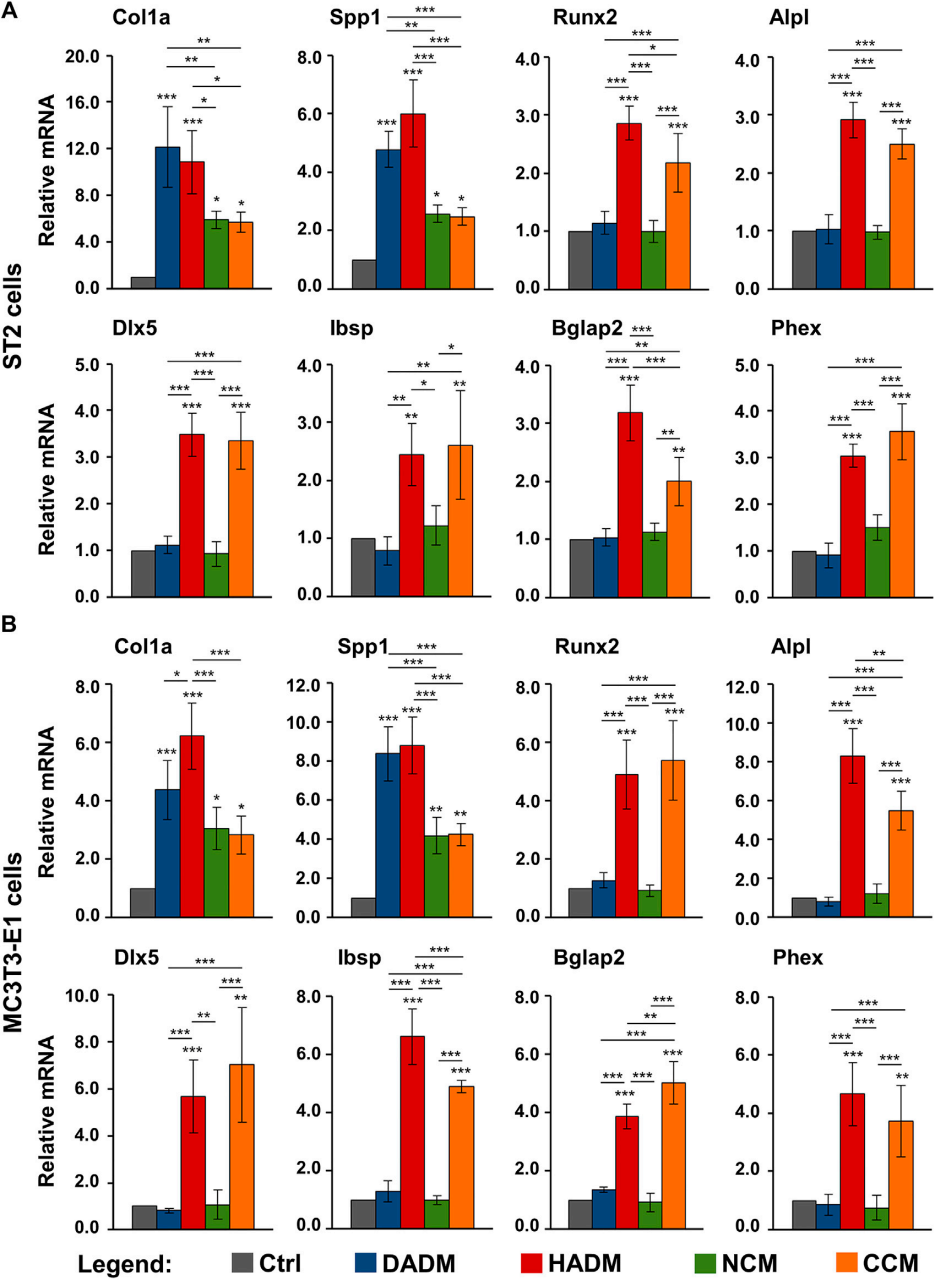
Increased expression of osteogenic differentiation markers in osteoprogenitor cells grown on HADM and CCM collagen-based matrices. ST2 **(A)** and MC3T3-E1 **(B)** cells were cultured in the absence of a collagen matrix (Ctrl) or on DADM, HADM, NCM, or CCM collagen-based matrices for 3 days before total cellular RNA was extracted, purified, and analyzed for the expression of Col1a1, Spp1, Runx2, Alpl, Dlx5, Ibsp, Bglap2, and Phex osteogenic markers by qRT-PCR. Values normalized to Gapdh are expressed relative to the values of control cells. Data represent means ± SD from four independent experiments performed with each of the two cell lines. Significant differences to the respective controls unless otherwise indicated, ***p < 0.001, **p < 0.01, *p < 0.05.

The qRT-PCR analyses showed that cells from each of the two lines grown on each of the four collagen-based scaffolds exhibited strongly induced expression of Col1a1 and Spp1 mRNAs above the expression levels detected in the respective control cells (Figure 5). The effects of DADM and HADM on the expression of Col1a and Spp1 in ST2 cells (Figure 5A) as well as on the expression of Spp1 in MC3T3-E1 cells (Figure 5B) were comparable and significantly (*p* < 0.05) better pronounced than the effects of NCM and CCM. Interestingly, HADM and CCM caused a strong (*p* < 0.01) upregulation of early, intermediate and late osteogenic differentiation markers whereas DADM and NCM had no effect on these transcripts. In isolated cases, the pro-osteogenic effects of HADM appeared superior compared to CCM. This was evident for the expression of Runx2 and Bglap2 in ST2 cells (Figure 5A) as well as for the expression of Alpl and Ibsp in MC3T3-E1 cells (Figure 5B). In contrast, the Bglap2 transcript in MC3T3-E1 cells was significantly (*p* < 0.01) better induced by CCM compared to HADM (Figure 5B).

These results suggest a stimulating effect of all investigated collagen-based scaffolds on the early stages of osteogenic differentiation, namely production of ECM that will later enable mineral deposition. Among the four investigated matrices, only HADM and CCM may be able to contribute to the osteogenesis progression by triggering the expression of osteogenic factors characterizing the advanced differentiation stages.

### Enhancing Effect of Collagen-Based Matrices Biofunctionalized With Enamel Matrix Derivative or Recombinant Bone Morphogenetic Protein-2 on the Expression of Osteogenic Differentiation Markers in Osteoprogenitor Cells

In the light of a limited modulation of the osteogenic process by only two of the four investigated collagen-based matrices, we investigated whether a short, clinically relevant coating of the scaffolds with either EMD or rBMP-2 can positively influence the osteogenic process. Earlier studies identified TGF-β1 in EMD by immunoassays (Stähli et al., 2014) and had shown that TGF-β-like activity can be passively released from EMD-coated collagen products (Stähli et al., 2016). To ensure proper technical performance of the coating experiments, on day 3, we measured the amounts of TGF-β1 and BMP-2 released in culture supernatants of cells cultured on EMD- and BMP-2-coated collagen matrices, respectively, by using ELISAs. The TGF-β1 was in the range of 2,140 ± 230 pg/ml ÷ 3,168 ± 220 pg/ml, and BMP-2 was in the range of 660 ± 55 pg/ml ÷ 940 ± 42 pg/ml.

**FIGURE 6:**
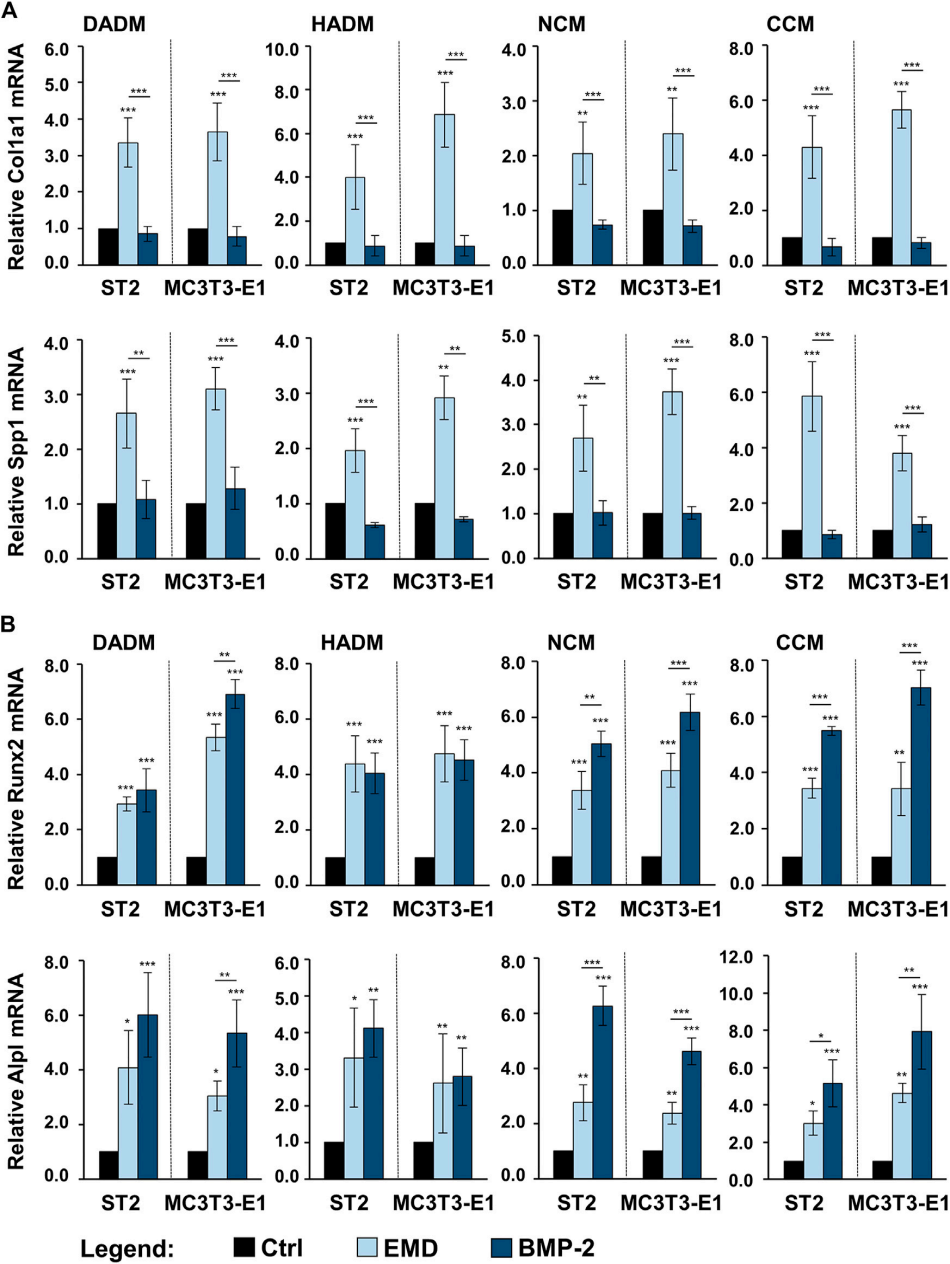
Enhancing effect of collagen-based matrices biofunctionalized with EMD or rBMP-2 on the expression of genes characterizing the early stages of osteogenic differentiation. Each of the two osteoprogenitor cell lines, ST2 and MC3T3-E1, were cultured on DADM, HADM, NCM, or CCM collagen-based matrices under three different conditions: 1) control condition (Ctrl), consisting of cells grown on native/uncoated matrices, 2) cells grown on matrices coated with EMD, and 3) cells grown on matrices coated with rBMP-2. For conditions 2) and 3), collagen matrices were coated for 10 min at room temperature in serum-free DMEM containing 1 mg/ml of EMD or 100 ng/ml of rBMP-2, respectively, followed by extensive wash of the matrices as described in the Materials and Methods section. Cells were grown under the above listed conditions for 3 days before total RNA was extracted and analyzed by qRT-PCR for the expression of Col1a1 and Spp1 **(A)**, Runx2 and Alpl **(B)**. Values normalized to Gapdh are expressed relative to the values of the respective control cells. Means ± SD from four independent experiments performed with each of the two cell lines and significant differences to the control unless otherwise indicated, ***p < 0.001, *p < 0.01, *p < 0.05 are shown.

In comparison with control conditions consisting of ST2 or MC3T3-E1 cells grown on the respective native/uncoated matrices, the expression levels of Col1a1 and Spp1 were significantly (*p* < 0.01) upregulated in osteoprogenitors cultured on all EMD-coated collagen matrices (Figure 6A). No effect of rBMP-2 was observed on the expression of these two genes. The expression of the early osteogenic marker genes Runx2 and Alpl was significantly (*p* < 0.05) induced by both EMD and rBMP-2 on each of the collagen-based matrices with a trend of a better pronounced effect of rBMP-2 in both ST2 and MC3T3-E1 cells cultured on NCM or CCM as well as in MC3T3-E1 cells cultured on DADM (Figure 6B).

**FIGURE 7:**
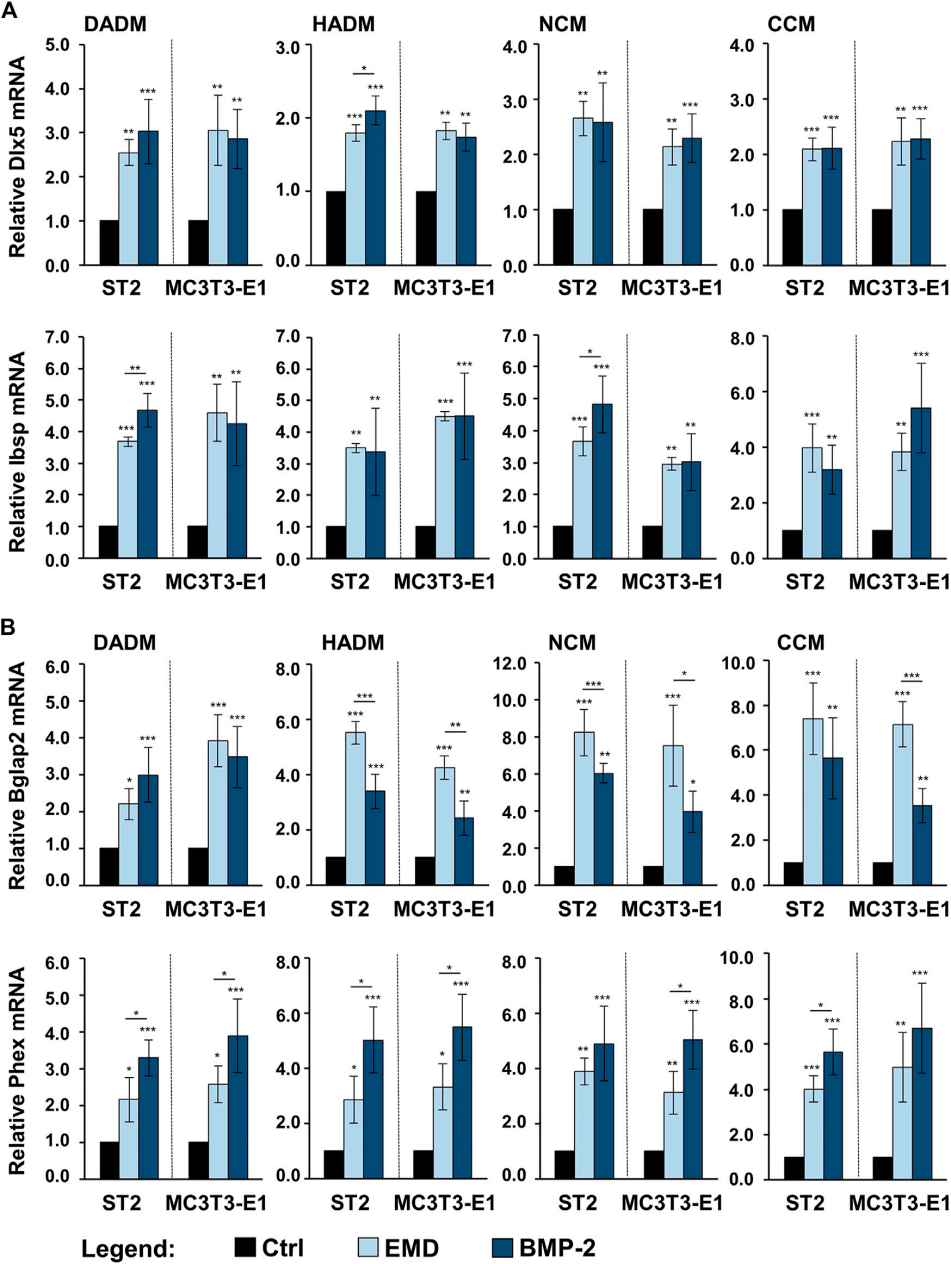
Enhancing effect of collagen-based matrices biofunctionalized with EMD or rBMP-2 on the expression of genes characterizing intermediate and late stages of osteogenic differentiation. Each of the two osteoprogenitor cell lines, ST2 and MC3T3-E1, were cultured on DADM, HADM, NCM, or CCM collagen-based matrices under three different conditions: 1) control condition (Ctrl), consisting of cells grown on native/uncoated matrices, 2) cells grown on matrices coated with EMD, and 3) cells grown on matrices coated with rBMP-2. For conditions 2) and 3), collagen matrices were coated for 10 min at room temperature in serum-free DMEM containing 1 mg/ml of EMD or 100 ng/ml of rBMP-2, respectively, followed by extensive wash of the matrices as described in the Materials and Methods section. Cells were grown under the above listed conditions for 3 days before total RNA was extracted and analyzed by qRT-PCR for the expression of Dlx5 and Ibsp **(A)**, Bglap2 and Phex **(B)**. Values normalized to Gapdh are expressed relative to the values of the respective control cells. Means ± SD from four independent experiments performed with each of the two cell lines and significant differences to the control unless otherwise indicated, ***p < 0.001, *p < 0.01, *p < 0.05 are shown.

In comparison with the respective uncoated matrices, all four matrices coated with either EMD or rBMP-2 were able to cause prominent upregulation of the intermediate and late osteogenic markers Dlx5, Ibsp, Bglap2, and Phex in each of the two cell lines (Figures 7A,B). The induction was in the range of 1.7–8.2-fold (*p* < 0.05). Interestingly, whereas on some of the scaffolds Dlx5, Ibsp, and Phex transcripts were characterized with a higher induction caused by rBMP-2 compared to EMD, the expression of Bglap2 mRNA was significantly better induced by EMD applied as a coating to HADM, NCM, and CCM. However, no clear pattern of better functionalization of the collagen matrices with one or the other bioactive substance could be identified.

The observed changes in the osteogenic marker gene expression indicate preserved biological activity of EMD and rBMP-2 adsorbed and released from each of the investigated collagen-based matrices as well as a clear stimulatory effect of each of the two substances on the osteogenic differentiation of the two osteoprogenitor lines. Whereas BMP-2 did not influence the expression of genes encoding bone matrix proteins, the effect of EMD was ubiquitous and spread over the entire range of genes regulating the osteogenic process.

## Discussion

Collagen-based biomaterials are shown to have advantages over other biomaterials and are therefore used in various tissue-engineering applications (Patil and Masters, 2020). Since collagen is the most abundant protein in the human body and a major component of bone and periodontal connective tissue, it appears chemotactic for various cell types (Farndale et al., 2004; Rothamel et al., 2004; Thibault et al., 2007; Lin et al., 2020), in addition to its prominent role in coagulum formation (Farndale et al., 2004; Kumar et al., 2014; Asparuhova et al., 2021) and angiogenesis at wounded sites (Twardowski et al., 2007). Clinically, collagen-based scaffolds are mostly utilized for guided tissue regeneration and soft tissue augmentation (Pabst and Kämmerer, 2020). The aim of the current study was to investigate the biocompatibility of different collagen-based scaffolds in cultures of mesenchymal stromal and pre-osteoblastic cells as well as to evaluate their potential to induce osteogenic cell differentiation *in vitro*. To the best of our knowledge, only one of the four matrices, namely the glycated CCM, has been previously tested in the context of osteogenesis, more specifically for restoring lost tissue volume of a deficient ridge (Smidt et al., 2019). Therefore, the current study appears to be the only one comparing the effects of the four different scaffolds on the behavior of cells involved in hard tissue regeneration.

**FIGURE 8:**
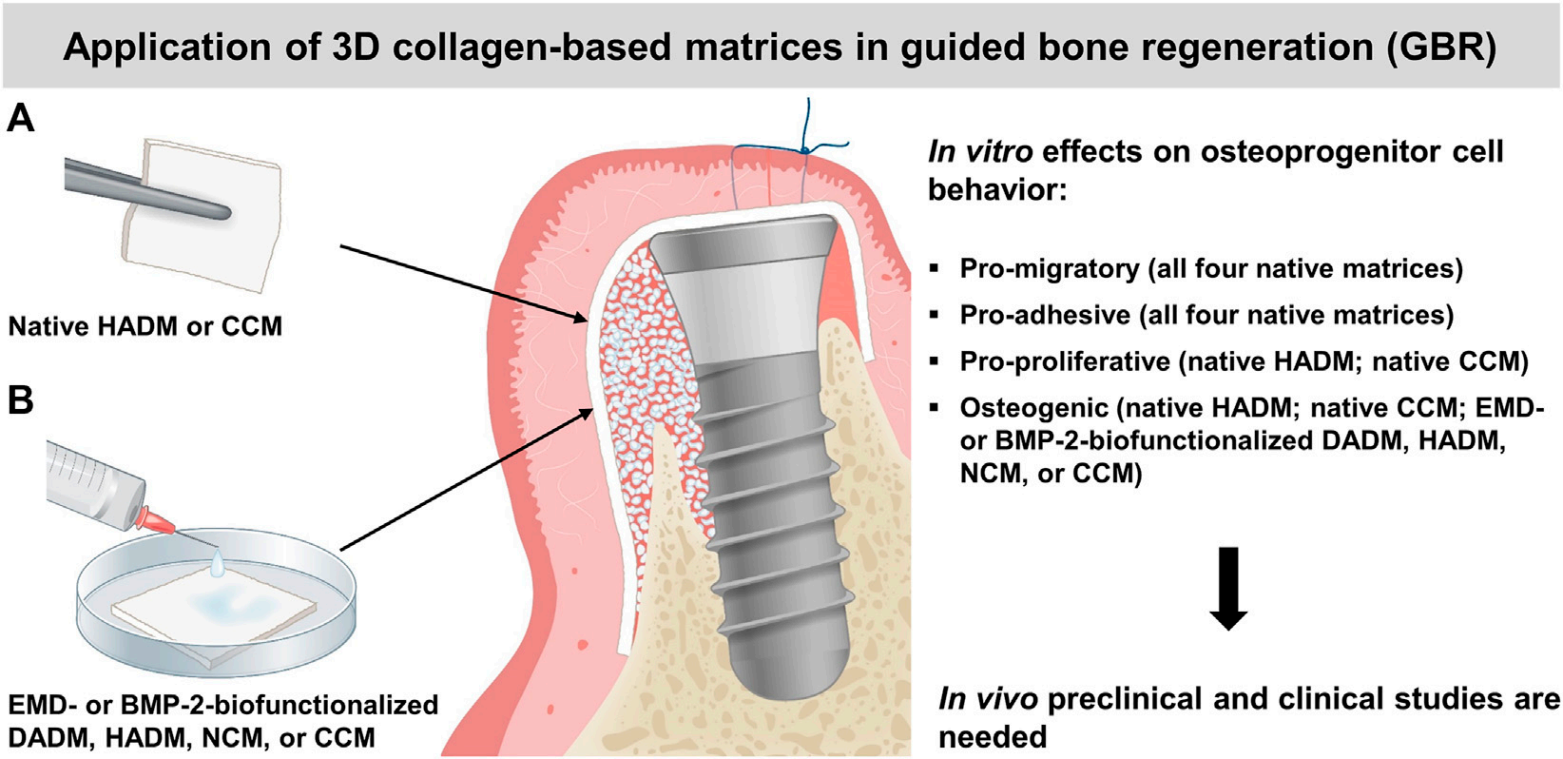
Summary of the *in vitro* results and suggested clinical application of the investigated 3D collagen-based matrices in guided bone regeneration (GBR). A schematic presentation of GBR with the use of autogenous bone or bone substitute material (white granules) for bone augmentation and a collagen matrix (pointed by arrows) as a barrier. The selective ingrowth of bone-forming cells into a bone defect site may be improved by the osteoinductive properties of the native HADM or CCM **(A)**, or the EMD- or BMP-2-biofunctionalized DADM, HADM, NCM or CCM **(B)**. The clinical application of the matrices in GBR is supported by their positive effects on the migratory, adhesive, proliferative, and differentiation properties of osteoprogenitor cells observed *in vitro*. The suggested application needs to be proved in future *in vivo* pre-clinical and clinical studies.

The osteogenic process is characterized by recruitment of osteoprogenitor cells, their attachment, growth, and differentiation into mature osteoblasts (Teti, 2011). Following the sequence of events accompanying the osteogenesis, our data have clearly demonstrated increased migratory, adhesive, proliferative, and osteogenic properties of mesenchymal stromal ST2 and pre-osteoblastic MC3T3-E1 cells grown on each of the four (for migration and adhesion) or on some (for proliferation and osteogenesis) matrices. Indeed, while all investigated 3D matrices exhibited favorable effects on the motility and attachment of the two osteoprogenitor cell lines, only the hydrated matrix of new generation, HADM and the crosslinked matrix, CCM were able to induce significant cell proliferation and to boost the expression of differentiation markers characterizing both early and late stages of the osteogenesis. However, the osteogenic potential of the other two matrices, DADM and NCM, was significantly boosted by EMD and rBMP-2 applied as a coating to the biomaterials. Taken together, the obtained results suggest an application of the 3D collagen-based matrices in guided bone regeneration (GBR). A summary of the results and suggested clinical application is depicted in Figure 8.

Interestingly, often chemically-induced crosslinking, e.g., the intra- and/or intermolecular crosslinking of collagen molecules with glutaraldehyde, has been proposed as a possible reason for a decreased biocompatibility of collagen-based materials (Marinucci et al., 2003; Adamiak and Sionkowska, 2020). In our study, the sugar-crosslinked CCM, which has been generated by a crosslinking method resembling the naturally occurring glycation process in mammalian cells, did not show any signs of reduced biocompatibility. On contrary, both the mesenchymal stromal and pre-osteoblastic cells showed increased proliferative rates on the CCM matrix that were comparable to the cell growth rates on the non-crosslinked HADM and superior compared with the natural NCM and DADM matrices. The impact of the four collagen-based matrices on the migratory, adhesive, and proliferative properties of ST2 and MC3T3-E1 cells, with a better pronounced pro-proliferative effect of HADM and CCM, resembled the impact of the matrices on the functionality of primary human periodontal ligament cells and oral fibroblasts investigated in a recently published study (Lin et al., 2020). This suggests that the difference in the potency of the four matrices to induce changes in the cellular behavior is not cell-specific but rather dependents on their physicochemical characteristics. Numerous investigations have demonstrated that the matrix composition, pore size and degree of porosity, and surface motifs involved in the cell recognition and cell-matrix interactions are in the basis of the differential behavior of cells grown on the different matrices (Stevens and George, 2005; Yeung et al., 2005; Chen et al., 2013; Rodina et al., 2016; Rodina et al., 2017). It has been shown that mesenchymal stem cells are prone to undergo osteospecific differentiation and functional bone tissue formation when cultured on topographies that increase the focal adhesion frequency and reinforcement (Biggs et al., 2009; Sjöström et al., 2009; Biggs and Dalby, 2010). Whereas fibroblasts prefer smooth or finely textured surfaces, osteoprogenitor cells attach better to rough or textured porous surfaces that would also enhance mineralization at the advanced stages of the osteoblast differentiation (Bowers et al., 1992; Rausch et al., 2021). Topographical analyses of the 3D matrices as well as investigations of the cell-matrix interactions in a direct way, by means of microscopy, have not been performed in the current investigation and deserve special attention.

Furthermore, focal adhesion reinforcement has been directly correlated with the expression of the transcription factor Runx2 as a master regulator of osteogenic marker gene expression (Salasznyk et al., 2007; Gordon et al., 2009). Upregulation of Runx2 expression has been documented in mesenchymal populations cultured on a variety of next generation biomaterials including 3D fibrous scaffolds (Woo et al., 2007), nanostructures (Mendonça et al., 2009), biofunctionalized titanium (Lim et al., 2009), and hydroxyapatite/tricalcium phosphate scaffolds (Sun et al., 2008). In our study, Runx2 as well as Alpl, Dlx5, Ibsp, Bglap2, and Phex transcripts were all significantly induced in osteoprogenitors cultured on native/non-functionalized HADM and CCM matrices only. This suggests that among the four investigated matrices, native HADM and CCM carry the greatest osteoinductive capacity. It remains to be elucidated whether native, unmodified HADM and CCM would have the ability to ossify when placed in proximity to bone *in vivo*. It is well known that collagen itself has a limited ability to induce apatite formation. Therefore, we investigated the possibility to use bioactive substances such as EMD, known to exhibit growth factor activities (Wyganowska-Świątkowska et al., 2015), or the highly osteogenic BMP-2 (Wozney et al., 1988) for biofunctionalization of the investigated biomaterials.

Recombinant growth factors are generally characterized with short half-lives, instability and fast degradation rates when applied in solution, side effects, and poor cost-effectiveness (Bowen-Pope et al., 1984; Anusaksathien and Giannobile, 2002; Carreira et al., 2014; Rocque et al., 2014). In a recent study, we have examined the adsorption and release of EMD and rBMP-2 from the four investigated collagen-based matrices (Nica et al., 2020). BMP-2 was characterized with relatively low release from all four matrices during the entire 13-day test period and several time points at which a burst release was observed. Based on findings demonstrating that TGF-β-like activity can be passively released from EMD-coated collagen products (Stähli et al., 2016), the EMD release kinetics was investigated by the means of TGF-β1 release and demonstrated a burst release within 24 h from HADM, DADM, NCM, and within 3 days from CCM, followed by a sustained slow release over 13 days. Both types of release kinetics were suggested as advantageous for the slow process of bone regeneration following implant placement or periodontal reconstruction. These findings prompted us to choose namely EMD and rBMP-2 as coatings in the present study.

EMD is an extract from fetal teeth composed of a mixture of enamel matrix proteins. Amelogenin proteins, including their enzymatically cleaved and alternatively spliced fragments, dominate this protein mixture by more than 90% (Grandin et al., 2012). The ability of EMD to regulate osteoblast proliferation and differentiation has been described as cell-specific and dependent on the stage of cell differentiation. More specifically, EMD has been shown to stimulate proliferation in the early stages of osteoblastic maturation and to enhance osteogenic differentiation in committed osteoblasts only (Schwartz et al., 2000). In our study, we have not detected any inverse relation between differentiation triggered by EMD-coated collagen matrices in the mesenchymal stromal ST2 and pre-osteoblastic MC3T3-E1 cells, likely due to the very close differentiation status of the two cell lines. Moreover, in agreement with earlier studies showing that EMD significantly upregulates Col1 (Du et al., 2005; Mrozik et al., 2012), Spp1 (Rincon et al., 2005), Alpl (Du et al., 2005; Fukae et al., 2006; Nagano et al., 2006), and Bglap2 (Iwata et al., 2002; Du et al., 2005) gene expression, we have demonstrated a stimulatory effect of EMD-coated matrices on the expression of the above listed genes. Indeed, various *in vitro* studies reported on the capacity of EMD to induce osteogenic gene expression in alveolar bone proper-derived stem cells (Fawzy El-Sayed et al., 2014) and dental follicle cells (Hakki et al., 2001) as well as to increase collagen, fibronectin, and TGF-β1 production in periodontal ligament cells (Van der Pauw et al., 2000). In addition to these *in vitro* studies, a number of clinical studies have reported a prominent regenerative effect of EMD in treating intrabony and furcation defects (Velasquez-Plata et al., 2002; Windisch et al., 2002; Donos et al., 2003; Sculean et al., 2003; Sculean et al., 2004; Losada et al., 2017).

BMP-2, on the other hand, belongs to the BMP subgroup of the TGF-β superfamily of proteins. The BMPs were first identified as factors able to induce ectopic bone formation *in vivo* (Wozney et al., 1988). Numerous studies on BMP-2 have shown that it strongly enhances the expression of osteogenic markers in cultures of bone marrow-derived stromal cells (Thies et al., 1992; Rickard et al., 1994; Asparuhova et al., 2018), myoblasts (Katagiri et al., 1994), alveolar bone proper-derived stem cells (Fawzy El-Sayed et al., 2014), and dental pulp stem cells (Hrubi et al., 2018). However, treatment of cells with BMP-2 have resulted in various outcomes and its osteogenic effects have appeared strongly dependent on the cell type, the applied dose, the treatment duration, and the local microenvironment, namely the presence of other osteoinductive molecules (Zhao et al., 2003; Hrubi et al., 2018). In this respect, it was not surprising that we observed no effect of BMP-2-coated matrices on the expression of genes encoding bone matrix proteins (Col1a1 and Spp1) but strongly induced expression of genes encoding early (Runx2 and Alpl), intermediate (Dlx5, Ibsp, and Bglap2), and late (Phex) osteogenic differentiation markers. On the one hand, the differential effects of EMD- and rBMP-2-coated matrices on the expression of Col1a1 and Spp1 transcripts can be explained by the proven TGF-β1 activity of EMD (Stähli et al., 2014; Wyganowska-Świątkowska et al., 2015; Stähli et al., 2016). TGF-β1 stimulates the synthesis of ECM proteins such as collagen, osteopontin, osteonectin, fibronectin, and integrins, and inhibits matrix degradation by stimulating the production of protease inhibitors and suppressing the production of proteases (Roberts et al., 1988). On the other hand, the observed similarities in the effects of EMD- and rBMP-2-coated matrices on the expression of the rest of osteogenic marker genes can be attributed to an existing relation between EMD and BMPs. It has been reported that 1) EMD upregulates the endogenous cellular production of BMPs (Parkar and Tonetti, 2004), and 2) EMD can be contaminated with trace amounts of active BMP-2 during the manufacturing process (Saito et al., 2008; Johnson et al., 2009).

The strategy of combining the investigated 3D collagen-based matrices with bioactive substances has the potential to enhance the bone regenerative process and to shed further light onto the underlying mechanisms of bone regeneration. In recent years, collagen-based matrices have been loaded not only with diverse bioactive substances but also with various cell types, drugs such as bisphosphonates, or vectors/nucleic acids encoding growth factors (Zhang et al., 2018). However, a composite collagen-based scaffold with ideal physicochemical and biological properties is still not available. Moreover, *in vivo* pre-clinical and clinical studies validating the utility of engineered composite scaffolds are largely missing. *In vitro* cell culture investigations carry the inherited limitation of testing biomaterials in the context of specific cell lines, which often do not fully represent the primary cells and are placed outside of their natural environment (in the case of two-dimensional culture models).

Within the limitations of the current *in vitro* study, it can be concluded that two of the four investigated collagen-based 3D scaffolds, namely the HADM and CCM, have the potential to be applied in GBR procedures (Figure 8A), in addition to their well-documented use as soft tissue substitute materials. With their pro-migratory, pro-adhesive, pro-proliferative, and osteogenic potential, HADM and CCM may allow osteoprogenitor cells to populate the protected space and to progress further into the differentiation process. Moreover, the presented study extends on the possibility to transfer osteoinductive properties onto the osteoconductive 3D collagen scaffolds by their short-term, clinically relevant pre-activation with EMD or rBMP-2 (Figure 8B). Such biofunctionalization may appear an optimal treatment modality for bone defects in periodontal and implant surgery.

## Data Availability

The original contributions presented in the study are included in the article/Supplementary Material, further inquiries can be directed to the corresponding author.
