# Supplementary material for: Positive Effects of Three-Dimensional Collagen-Based Matrices on the Behavior of Osteoprogenitors
Source: Front Bioeng Biotechnol. 2021 Jul 21;9:708830. doi: 10.3389/fbioe.2021.708830 (PMC8334008; doi:10.3389/fbioe.2021.708830)
Supplement: Supplementary file 1 [file DataSheet1.PDF]

Supplementary Material

Supplementary Table 1. Primer sequences for proliferative marker genes.

Gene symbol	Primer pair (fwd/rev)
Mybl2	5′-GCCCATAAAGTCCTGGGTAAC-3'<br>5′-CACAGCATTGTCCGTCCTC-3'
Bub1	5′-TCCTGTAAGTGGCCAGTCATT-3'<br>5′-TGAATTCATGAACACTTTGATTCAC-3'
Plk1	5′-TTGTAGTTTTGGAGCTCTGTCG-3'<br>5′-CAGTGCCTTCCTCCTCTTGT-3'
Mki67	5′-GCTGTCCTCAAGACAATCATCA-3'<br>5′-GGCGTTATCCCAGGAGACT-3'
Pcna	5′-GAATCCCAGAACAGGAGTACAG-3'<br>5′-GACAACTTAATGTTCCCATTGCC-3'
Ccne1	5′-GAAGGTCTCAGGTTATCAGTGG-3'<br>5′-GTGTGGGTCTGGATGTTGTG-3'
Ccnd1	5′-AGAAGTGCGAAGAGGAGGTC-3'<br>5′-AGTTCCATTTGCAGCAGCTC-3'
Ccnb1	5′-GTGAATGGACACCAACTCTG-3'<br>5′-ACAGTCATGTGCTTTGTGAG-3'
Gapdh*	5′-CTTGTGCAGTGCCAGCCTC-3'<br>5′-GCCGTGAGTGGAGTCATACTG-3'

*reference gene used for normalization in all qPCR analyses

Supplementary Table 2. Primer sequences for adhesive marker genes.

Gene symbol	Primer pair (fwd/rev)
Fn1	5′-GCCACCATTACTGGTCTGGA-3'<br>5′-CCGCCTAAAGCCATGTTCCT-3'
Vcl	5′-TCTCGCACCTGGTGATTATGC-3'<br>5′-TGAACAGTCTCTTTTCCAACCC-3'
Cd44	5′-CACCATTGCCTCAACTGTGC-3'<br>5′-TTGTGGGCTCCTGAGTCTGA-3'
Icam1	5′-CCCACGCTACCTCTGCTC-3'<br>5′-GATGGATACCTGAGCATCACC-3'

Supplementary Table 3. Primer sequences for osteogenesis marker genes.

Gene symbol	Primer pair (fwd/rev)
Col1a1	5′- CCGGAAGAATACGTATCACCA-3'<br>5′- TCTGGGAAGCAAAGTTTCCT-3'
Spp1	5′-GGAAACCAGCCAAGGTAAGC-3'<br>5′-TGCCAATCTCATGGTCGTAG-3'
Runx2	5′- AGGGACTATGGCGTCAAACA-3'<br>5′- GGCTCACGTCGCTCATCTT-3'
Alpl	5′- GCAACTCCATCTTTGGTCTG-3'<br>5′- GTTGTTGTGAGCGTAATCTACC-3'
Dlx5	5′- GTCCCAAGCATCCGATCCG-3'<br>5′- GCGATTCCTGAGACGGGTG-3'
Ibsp	5′- GGTCTTTAAGTACCGGCCAC-3'<br>5′- CGTTTGAAGTCTCCTCTTCCTC-3'
Bglap2	5′- CACCTAGCAGACACCATGAG-3'<br>5′- TGGACATGAAGGCTTTGTCAG-3'

Phex	5′- GAAAGGGGACCAACCGAGG-3'	
	5′- AACTTAGGAGACCTTGACTCACT-3'	
